# Growth characteristics of primary M_7_C_3_ carbide in hypereutectic Fe-Cr-C alloy

**DOI:** 10.1038/srep32941

**Published:** 2016-09-06

**Authors:** Sha Liu, Yefei Zhou, Xiaolei Xing, Jibo Wang, Xuejun Ren, Qingxiang Yang

**Affiliations:** 1 State Key Laboratory of Metastable Materials Science & Technology, Yanshan University, Qinhuangdao 066004, P. R. China; 2 College of Mechanical Engineering, Yanshan University, Qinhuangdao 066004, P. R. China; 3 School of Engineering, Liverpool John Moores University, Liverpool L3 3AF, UK

## Abstract

The microstructure of the hypereutectic Fe-Cr-C alloy is observed by optical microscopy (OM). The initial growth morphology, the crystallographic structure, the semi-molten morphology and the stacking faults of the primary M_7_C_3_ carbide are observed by scanning electron microscopy (SEM) and transmission electron microscopy (TEM). The in-suit growth process of the primary M_7_C_3_ carbide was observed by confocal laser microscope (CLM). It is found that the primary M_7_C_3_ carbide in hypereutectic Fe-Cr-C alloy is irregular polygonal shape with several hollows in the center and gaps on the edge. Some primary M_7_C_3_ carbides are formed by layers of shell or/and consist of multiple parts. In the initial growth period, the primary M_7_C_3_ carbide forms protrusion parallel to {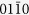} crystal planes. The extending and revolving protrusion forms the carbide shell. The electron backscattered diffraction (EBSD) maps show that the primary M_7_C_3_ carbide consists of multiple parts. The semi-molten M_7_C_3_ carbide contains unmelted shell and several small-scale carbides inside, which further proves that the primary M_7_C_3_ carbide is not an overall block. It is believed that the coalescence of the primary M_7_C_3_ carbides is ascribed to the growing condition of the protrusion and the gap filling process.

he Fe-Cr-C system has been investigated many times over the years. The Fe-Cr-C system contains kinds of carbides such as M_23_C_6_, M_7_C_3_ and M_3_C_2_^1^. W. Tofaute *et al*. dealt with the 850 °C and 1560 °C isothermal sections of Fe-Cr-C system^2^,^3^ and R. S. Jackson dealt with the 900 °C isothermal section^4^, which found that the M_23_C_6_ carbides are in equilibrium with both austenite and ferrite, whereas the M_3_C and M_7_C_3_ carbides are in equilibrium with austenite only. Hypereutectic Fe-Cr-C alloy is a kind of typical wear-resistant material^5^,^6^,^7^. Its wear-resistance is closely related to the transition-metal carbides, especially the M_7_C_3_ (M=Fe, Cr) carbide^8^,^9^,^10^,^11^. X. H. Tang *et al*.^12^ have revealed that the mass fraction of the primary M_7_C_3_ carbides is increased with increasing C content and the wear-resistance is improved accordingly. C. M. Lin *et al*.^13^ have proved that the orientation of the primary M_7_C_3_ carbide can also influence the wear-resistance of the hypereutectic Fe-Cr-C alloy. Refs 14, 15, 16, 17 found that the refined primary M_7_C_3_ carbides via fast-cooling can obviously increase the hardness of the hypereutectic Fe-Cr-C alloy so that its wear-resistance and corrosion-resistance are improved accordingly.

Nowadays, the researches of the primary M_7_C_3_ carbide in the hypereutectic Fe-Cr-C alloy are mainly focused on its performance. While the growth mechanism of the primary M_7_C_3_ carbide is investigated rarely. A. Leško *et al*.^18^ have put forward a theory on the growth of M_7_C_3_ carbide in the basis of the dissolved screw dislocation core. It held that the inherent screw dislocations on the basal-plane of the hexagonal M_7_C_3_ carbide can form the revolving steps along the c-axis. The mobile atoms in the molten alloy can be easily adsorbed on the steps so that the M_7_C_3_ carbide preferentially grows along the c-axis. In the center of the growing carbide, the molten alloy is surrounded by the solidified carbide so that the internal hollow can be formed. S. Q. Ma *et al*.^19^ have put forward another outflank theory that the inherent stacking faults on the {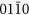} crystal planes can cause the growth of the M_7_C_3_ carbide along these planes. So the hexagon-shaped M_7_C_3_ carbide can be formed. However, it is found in many studies that the primary M_7_C_3_ carbide is irregular polygon shape with several hollows and some gaps on the edge, rather than the simple shape mentioned above^20^,^21^. These phenomena can be hardly explained by the two theories mentioned above.

In this paper, the initial growth morphology, the crystallographic structure, the semi-molten morphology and the stacking faults of the primary M_7_C_3_ carbide are investigated systematically on the basis of morphology observation and in-suit growth observation. By analyzing the results, the growth characteristics of the primary M_7_C_3_ carbide is proposed, which provides reasonable interpretation for the irregular polygonal shape of the primary M_7_C_3_ carbide with several hollows in the center and gaps on the edge.

## Results

### Morphology of primary M_7_C_3_ carbide

**Figure 1:**
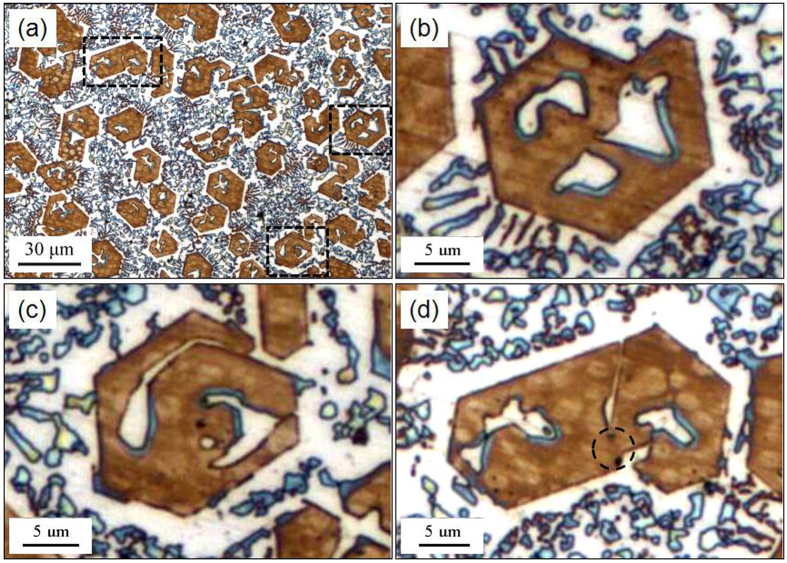
Morphology of the primary M_7_C_3_ carbides in the hypereutectic Fe-Cr-C alloy. (**a**) OM image of the as-welding Fe-Cr-C alloy; (**b**–**d**) magnifications of the typical primary M_7_C_3_ carbides.

Figure 1 shows the original as-welding morphology of the primary M_7_C_3_ carbides in the hypereutectic Fe-Cr-C alloy. Figure 1(a) is the OM image of the dyed specimen, in which the brown polygons are the cross-sections of the primary M_7_C_3_ carbides. Although the cross-sections of primary M_7_C_3_ carbides are not hexagon or hollow-hexagon shape, the included-angle between the adjacent edges of primary M_7_C_3_ carbides are 120°. Figure 1(b–d) show the magnifications of three typical primary M_7_C_3_ carbides. The primary M_7_C_3_ carbide in Fig. 1(b) contains three hollows in the center. The primary M_7_C_3_ carbide in Fig. 1(c) is formed by layers of non-closed shell. The primary M_7_C_3_ carbide in Fig. 1(d) consists of two visible parts and the right part exhibits a gap on the edge. The two parts contact in the position marked by the circle.

### Protrusion in primary M_7_C_3_ carbide

**Figure 2:**
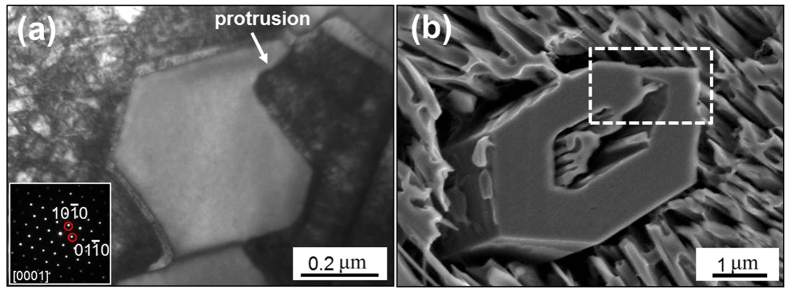
Protrusions of the primary M_7_C_3_ carbides in their initial growth period. (**a**) Bright field TEM image; (**b**) SEM image.

Figure 2(a,b) shows the initial growth morphologies of the primary M_7_C_3_ carbide via TEM and SEM respectively. It is found that the primary M_7_C_3_ carbide exhibits protrusion phenomenon in the initial growth period. Figure 2(a) is the bright field TEM image of the primary M_7_C_3_ carbide. Based on the selected area diffraction pattern (SADP), the primary M_7_C_3_ carbide is hexagonal structure and the zone axis is [0001]. The diameter of the carbide is about 0.4 μm. The carbide forms a protrusion parallel to the {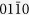} crystal plane. Figure 2(b) is the SEM image of the primary M_7_C_3_ carbide. The primary M_7_C_3_ carbide is non-closed hollow-hexagon shape, which is enclosed by layer of shell. Close inspection of the dashed box in Fig. 2(b) indicates that the carbide shell exhibits the phenomenon of “head” linked with “tail”. The obtuse “head” is similar with the carbide in Fig. 2(a) in size and shape, while the sharp “tail” is similar with the protrusion in Fig. 2(a).

### In-suit observation of primary M7C3 carbide growth process

**Figure 3:**
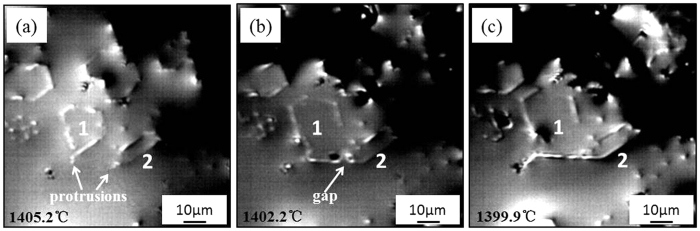
In-suit observations of the primary M7C3 carbides growth process.

Figure 3 shows the in-suit observations of the primary M_7_C_3_ carbide growth process. It can be found from Fig. 3(a) that the primary M_7_C_3_ carbides marked as 1 and 2 both form protrusions at 1405.2 °C. It can be found from Fig. 3(b) that at 1402.2 °C, the growth directions of the protrusions both revolve 120°, especially the protrusion in carbide 1. While the protrusions do not touch and a gap is existed between the two primary M_7_C_3_ carbides. It can be seen in Fig. 3(c) that the gap is filled and the two primary carbides form a bulky M_7_C_3_ carbide at 1399.9 °C.

### Crystallographic structure of primary M_7_C_3_ carbide

**Figure 4:**
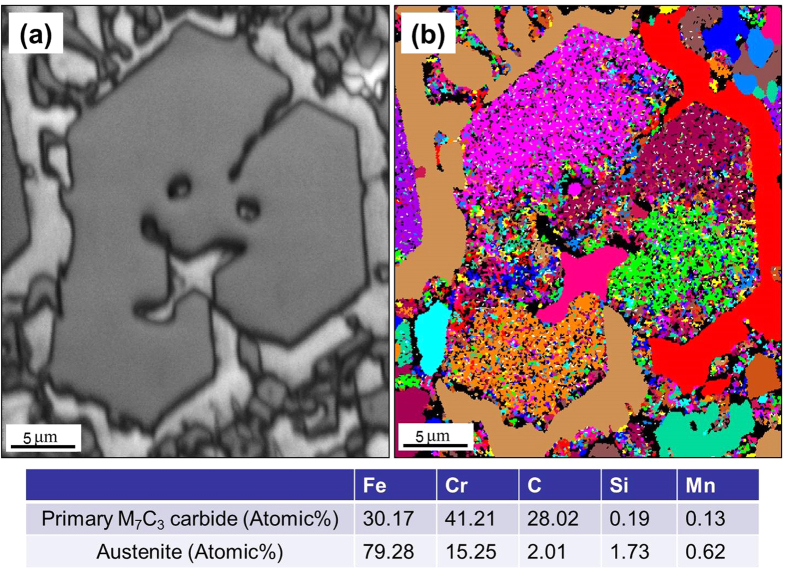
EBSD maps and compositions of the Fe-Cr-C alloy. (**a**) IQ map; (**b**) UGC map.

Figure 4 shows the EBSD maps and compositions of the Fe-Cr-C alloy. Figure 4(a) is the image-quality (IQ) map, in which the bulky gray polygon is the primary M_7_C_3_ carbide and the surrounding small gray pieces are the eutectic M_7_C_3_ carbides. Figure 4(b) is the unique grain color (UGC) map, in which the grains are simply colored to distinguish them from neighboring grains. In TSL OIM Analysis 7.0 software, the UGC map is the best way to see how the scanning points are grouped into grains. It groups the scanning points into different grains by identifying their orientations and the grains are colored differently. It can be found from Fig. 4(b) that the primary M_7_C_3_ carbide is not an overall block but consists of multiple parts. The table in Fig. 4 shows the compositions of primary M_7_C_3_ carbide and austenite respectively. The ratio of metal atoms to C atoms is 7:3, which agrees with the stoichiometry of M_7_C_3_ carbide. The Cr/Fe ratio is near 4:3, which is in accordance with our previous study by first-principles calculations that the Fe_3_Cr_4_C_3_ carbide is more stable than other M_7_C_3_ (M=Fe, Cr) carbides^22^. Compared with primary M_7_C_3_ carbide, austenite contains more Fe atoms but fewer Cr and C atoms.

## Discussion

**Figure 5:**
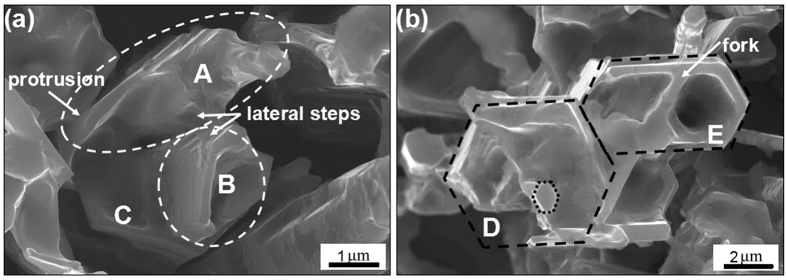
3D-morphologies of the primary M_7_C_3_ carbides in their initial growth period. (**a**) Three-part morphology; (**b**) two-part morphology.

Figure 5 shows the 3D-morphologies of the primary M_7_C_3_ carbide in the initial growth period after heat-treatment. Compared with the as-welding specimen in Fig. 1, the axial and radial dimensions of the primary M_7_C_3_ carbide are small so that the growth front of the carbide can be observed. The primary M_7_C_3_ carbide in Fig. 5(a) consists of three parts which are named as A, B, C respectively. The obtuse head of part A is prism shape and there forms a protrusion parallel to its lateral plane. Part B is non-closed hollow-hexagon shape, which is enclosed by carbide shell. Part A and part B are grown together and there exist visible steps on their lateral plane. Part C is placed below part A and part B. The dimension of part C is larger than those of part A and part B. Part A and part B are enclosed within part C and grown together with it. The primary M_7_C_3_ carbide in Fig. 5(b) consists of two parts, which are named as D, E respectively (the left bottom corner of part D is fallen during specimen preparation). Part D and part E are both hexagon shape with hollows or concave in the center, and they are also grown together. Part D is similar with part C, which also encloses a small carbide within it, as shown by the dotted hexagon. Part E is similar with part B, which is also non-closed hollow-hexagon shape enclosed by carbide shell. However, the carbide shell of part E exists a fork, where a branch extends along the original direction while the other branch revolves 120°. Compared to Figs 4 and 5 also proves that the primary M_7_C_3_ carbide is not an overall block but consists of multiple parts.

By comparing Figs 1 to 5, it can be found that they have many similarities. The primary M_7_C_3_ carbide in Fig. 1(b) is similar with the part E in Fig. 5(b), whose multiple hollows in the center is due to the folk and the revolving of the carbide shell. The primary M_7_C_3_ carbide formed by layers of shell in Fig. 1(c) and the primary M_7_C_3_ carbide composed of multiple parts in Fig. 1(d) can be seen from Fig. 5 too.

**Figure 6:**
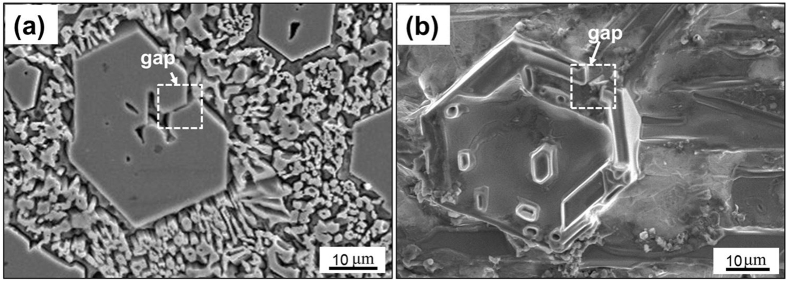
SEM images of the primary M_7_C_3_ carbide before (**a**) and after (**b**) semi-melting.

Figure 6 shows the SEM images of the primary M_7_C_3_ carbide before and after semi-melting. Figure 6(a) is the typical as-welding morphology of the primary M_7_C_3_ carbide, which contains several hollows in the center and a gap on the edge. The surrounding small pieces are the eutectic M_7_C_3_ carbides. Figure 6(b) is the semi-molten morphology of the primary M_7_C_3_ carbide. It can be found that the eutectic M_7_C_3_ carbides are all molten. While the primary M_7_C_3_ carbide is partially molten so that it is with concave surface rather than smooth ground surface as Fig. 6(a). The outer shell of the primary M_7_C_3_ carbide is similar with that in Fig. 6(a) in size and shape, and it also contains a gap. Figure 6(b) shows that the outer shell of the primary M_7_C_3_ carbide is not molten at all. Moreover, several unmelted small carbides exist within the primary M_7_C_3_ carbide, which is similar with Fig. 5. The unmelted outer shell and small carbides illustrate that the primary M_7_C_3_ carbide is not an overall block but consists of multiple parts, which is in accordance with Fig. 4(b).

**Figure 7:**
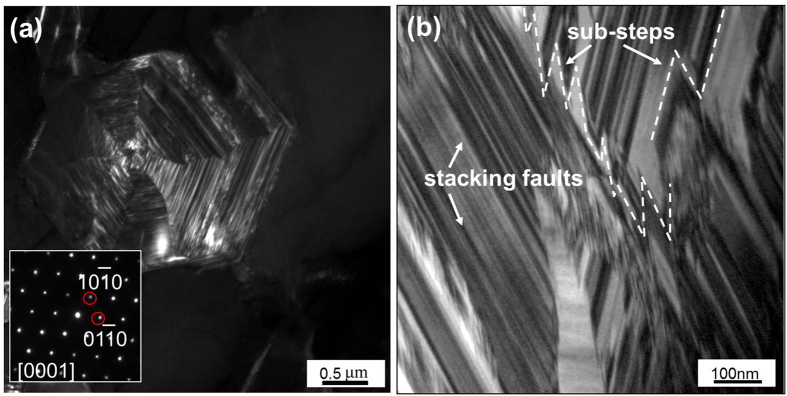
Stacking faults of the primary M_7_C_3_ carbides. (**a**) TEM dark field image; (**b**) magnification of the stacking faults.

Figure 7 shows the TEM images of the primary M_7_C_3_ carbide. Figure 7(a) is the dark field image, from which it can be found that there exist lots of stacking faults parallel to the {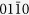} crystal planes. Figure 7(b) is the magnification of the stacking faults. It can be seen that the stacking faults form lots of sub-steps parallel to the {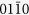} crystal planes. There are literatures have proved the appearance of the stacking faults with fault vector of 120° included-angle^23^,^24^,^25^,^26^. They have also proved the appearance of the sub-steps with a height of 1/2d{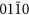} as shown in Fig. 7(b). A layer of mobile atoms will be absorbed on the sub-step and then another sub-step with the same height will be formed in the opposite side. The self-perpetuating steps are created constantly, which leads to the growth of M_7_C_3_ carbide along {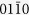} crystal planes^19^.

However, the diversity of the defects in the M_7_C_3_ carbide makes the difference in the growth rate along {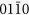} crystal planes, which forms the protrusion in Fig. 2(a). The defects at the protrusion are more complex so it may grow faster. Furthermore, defects such as screw dislocations may shift the growth direction of the M_7_C_3_ carbide. Because the included-angle of the {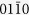} crystal planes is 120°, and the included-angle between the stacking fault and its fault vector is also 120°, the growth direction of the protrusion may revolve from a crystal plane in {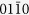} to another, as seen in Fig. 3. In this way, the M_7_C_3_ carbide grows into hollow-hexagon shape formed by layer of shell which can be seen in Fig. 2(b). And it can be deduced that the sharp tail in Fig. 2(b) is the growing-tip. On condition that the protrusion revolves partially while the other part remains the original direction, the carbide shell will fork, which is shown in Figs 1(b) and 5(b). Besides of the diversity of the defects, the impurities and cooling rate may also influence the growing condition of the protrusion. Alloy element Al in the welding material is employed to purify the molten alloy. When Al atoms are absorbed on the protrusion, the transport of Fe, Cr and C atoms through the M_7_C_3_/liquid interface will be suppressed, which prevents the growth of the protrusion. Therefore, the non-closed carbide shell in Fig. 1 can be seen. Meanwhile, the primary M_7_C_3_ carbide grows faster with higher cooling rate, which promotes the revolving of the protrusion. So the size of the primary M_7_C_3_ carbide will decrease as the cooling rate increases^27^.

**Figure 8:**
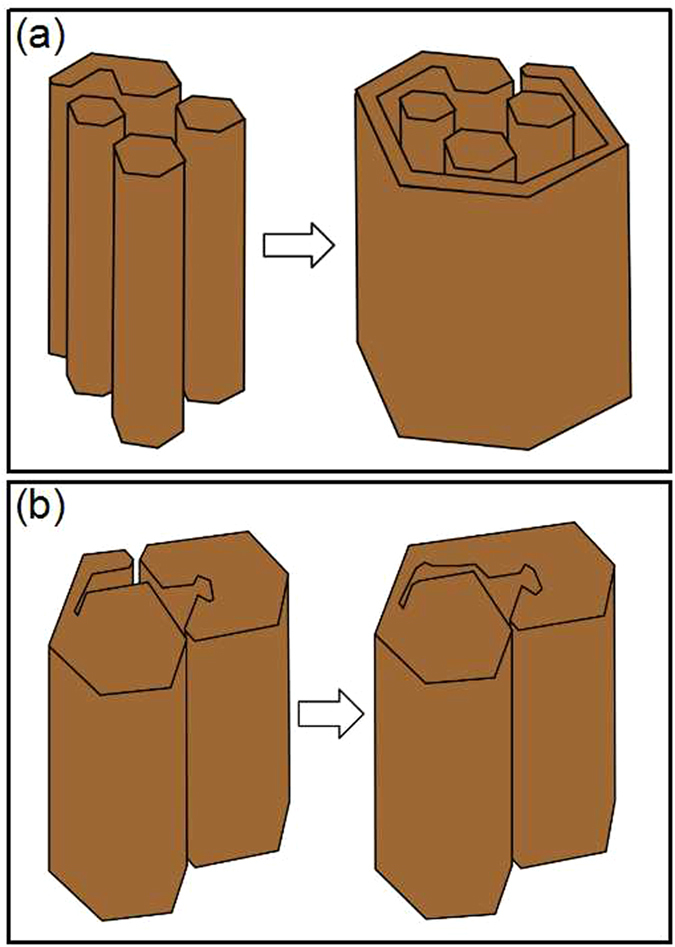
Schematic diagrams of the M_7_C_3_ carbides coalescence process.

As mentioned above, the primary M_7_C_3_ carbide is not an overall block but a combination of multiple parts. So the growth characteristics of the primary M_7_C_3_ carbide are schematically shown in Fig. 8. In the solidification process of the molten alloy, the primary M_7_C_3_ carbide precipitates at high temperature. Among the initially growing M_7_C_3_ carbides, some may form the carbide shell by revolving growth direction which is shown in Figs 2 and 3. The constantly growing and revolving protrusion may encloses some small carbides within the carbide shell inevitably, which is schematically shown in Fig. 8(a). Because the growth along the c-axis is caused by the revolving steps^18^, there exist lateral steps on the lateral plane which is shown in Fig. 5(a). The lateral steps can easily absorb the mobile atoms so that the gaps among the carbides can be filled. Ultimately, bulky M_7_C_3_ carbide is formed. Because the temperature of gap filling is lower than that required by primary M_7_C_3_ carbide precipitation, the bulky M_7_C_3_ carbide after semi-melting shows the morphology as seen in Fig. 6(b). Furthermore, the growth of the protrusions can also causes the coalescence of the adjacent M_7_C_3_ carbides, which is schematically shown in Fig. 8(b). The gaps among them can also be filled by absorbing mobile atoms on the lateral steps. Unlike the growth along the c-axis based on the steps, the gap filling process is dominated by the atomic diffusion and concentration. The primary M_7_C_3_ carbide is rich in C and Cr atoms, but poor in Fe atoms (shown in Fig. 4). In the coalescence process, the C and Cr atoms in the gaps are continuously consumed, which decreases the diffusions of C and Cr atoms toward the M_7_C_3_/liquid interface. It causes the gap filling process to end incompletely. Moreover, with the continuous absorption of the mobile atoms, their concentrations in the molten alloy are reduced accordingly. When the atomic concentrations are reduced to a certain degree, the coalescence process of the M_7_C_3_ carbides is finished. Therefore, there are always several hollows in the center and gaps on the edge of the primary M_7_C_3_ carbide. In conclusion, the morphologies of the primary M_7_C_3_ carbides (seen in Fig. 1) are ascribed to the growing condition of the protrusion and the gap filling process.

Based on the morphology, the in-suit growth process, the initial growth morphology, the crystallographic structure, the semi-molten morphology and the stacking faults of the primary M_7_C_3_ carbide, the following conclusions can be drawn:

- The primary M_7_C_3_ carbide in the hypereutectic Fe-Cr-C alloy shows irregular polygonal shape with several hollows in the center and gaps on the edge. Some primary M_7_C_3_ carbides are formed by layers of shell or/and consist of multiple parts.
- The growth along {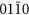} crystal planes has difference rates which forms the protrusion parallel to the {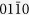} crystal planes. The constantly growing and revolving protrusion forms the M_7_C_3_ carbide shell and the sharp tail is the growing-tip. Some small carbides are enclosed within the carbide shell.
- The steps on the lateral planes of the primary M_7_C_3_ carbides can easily absorb the mobile atoms, so that the gaps among the adjacent carbides can be filled. Ultimately, bulky M_7_C_3_ carbide is formed. The growth of the protrusions can also cause the coalescence of the adjacent M_7_C_3_ carbides. The hollows in the center and gaps on the edge of the primary M_7_C_3_ carbide are ascribed to the end of the coalescence process.

## Methods

### Sample preparation

In this paper, the experimental material is hypereutectic Fe-Cr-C alloy prepared by hard-facing welding. The welding material was self-made flux-cored wires. The welding voltage and current were 24 V and 200 A respectively. The welding speed was 300mm/min. After welding, the alloy was sprayed with water for faster cooling and stress releasing. The composition of the hypereutectic Fe-Cr-C alloy was measured by Advant/p-381 X-ray fluorescence spectrometer and CS-8800 infrared carbon-sulfur analyzer, which showed that the composition was 26.3 wt.%Cr, 3.7 wt.% C, 1.0 wt.% Si, 0.3 wt.% Mn, 0.07 wt.% Al, 0.02 wt.% S, 0.03 wt.% P and Bal. Fe.

In order to keep the initial growth state of the primary M_7_C_3_ carbide, the hypereutectic Fe-Cr-C alloy was heated to 1500 °C and held for 20 minutes by the infrared heater equipped on the VL2000DX-SVF17SP confocal laser microscope (CLM) to ensure that the alloy is completely molten. The molten alloy was slowly cooled at 5 °C/min to 1410 °C where the primary M_7_C_3_ carbide starts to precipitate. Soon the alloy is sharply cooled. The temperature controlling accuracy of the infrared heater is ±0.1 °C. The cooling speed is −100 °C/s so that the initial growth state of the primary M_7_C_3_ carbide can be fixed.

The hypereutectic Fe-Cr-C alloy was also heated to 1500 °C by the infrared heater, and then the molten alloy was slowly cooled at 5 °C/min till the molten alloy was transformed into solid phase. The in-suit growth process of primary M_7_C_3_ carbide was observed by CLM.

Moreover, the hypereutectic Fe-Cr-C alloy was heated to 1400 °C and soon sharply cooled by the infrared heater to keep the semi-molten state of the primary M_7_C_3_ carbide.

### Characterization

The metallographic observation of the specimen was conducted by Axiovert 200 MAT optical microscopy (OM). Before the observation, the specimen was ground by SiC abrasive paper and polished by diamond abrasion paste. Then the specimen was dyed with 25% K3Fe(CN)_6_ + 7%NaOH + 68% H_2_O solution.

The secondary electron scanning and backscattered electron scanning were conducted by Hitachi S3400N field emission scanning electron microscopy (FESEM). Before the secondary electron scanning, the specimen was ground by SiC abrasive paper and polished by diamond abrasion paste. Then the specimen was deep-etched with 50% HCl + 50% C_2_H_5_OH solution. Before the backscattered electron scanning, the polished specimen was electropolished by ElectroMet 4 electropolisher with 85% C_2_H_5_OH + 10%HClO_4_ + 5% C_3_H_8_O_3_ solution. The backscattered electron scanning step was 0.2 μm. The electron backscattered diffraction (EBSD) results were analyzed by TSL OIM Analysis 7.0 software.

The transmission electron observation was conducted by JEM-2010 transmission electron microscopy (TEM). Before the observation, the foil specimen about 300 μm thick was ground by SiC abrasive paper to about 40 μm and then thinned by Gatan precision ion polishing system (PIPS). The results were analyzed by DigitalMicrograph 3.10.0 software.

## Additional Information

**How to cite this article**: Liu, S. *et al*. Growth characteristics of primary M_7_C_3_ carbide in hypereutectic Fe-Cr-C alloy. *Sci. Rep*. **6**, 32941; doi: 10.1038/srep32941 (2016).
